# From the Editor's desk

**DOI:** 10.4103/0974-1208.63112

**Published:** 2010

**Authors:** Kamini A Rao

**Affiliations:** Editor-in-Chief, Journal of Human Reproductive Sciences E-mail: kambacc@vsnl.com

The current issue of Journal of Human Reproductive Sciences addresses fertility preservation in young cancer patients in the review article. While gonadotoxic consequences of cancer therapy can be a daunting prospect for anybody, it is especially overwhelming for the young adolescents. Over the last several decades, survival rates for childhood cancer have steadily increased. With the overall cure rate for pediatric malignancies now approaching 80%, it has resulted in a major interest in long-term effects of cancer treatment and the possibilities for future fertility. If the pace of reproductive advances to date is any indicator of future successes, there is a reason to be hopeful that the advanced laboratory techniques developed for utilizing cryopreserved testicular tissue to restore fertility can be translated for use in human subjects and soon achieve the same success as that of ovarian tissue.

Advances in imaging techniques have enabled accurate diagnosis of uterine and adnexal disease, thus redefining the role of laparoscopy. Dr. Jayakrishnan and his team have successfully shown that laparoscopy in women with negative findings helps at least a fourth of the women to improve future fertility by treatment. Couples who fail to conceive with ovulation stimulation with IUI should be counseled that laparoscopy is helpful before proceeding to ART.

Dr. Ghaseem Saki and team have successfully demonstrated the deleterious effect of stress on animal semen quality such as the number of sperm, fertilization capacity, as well as motility of sperm.

Finally some unusual case reports can be interesting to read. One of them on congenital malformation of fetus in a pregnancy following spontaneous ovulation in a case of premature ovarian failure and another of symptomatic unilateral pleural effusion. A rare presentation of ovarian hyper-stimulation syndrome can also be an interesting reading. All in all, this issue has some pretty interesting articles which I hope you will enjoy.

There are four letters to the editor, of which one is by Hong Ye on an article that suggests that the degree of estradiol decline in early and mid luteal phase does not seem to have a harmful effect on IVF/ICSI outcome. The role of E2 in the luteal phase has been the subject of a long and ongoing controversy and while this retrospective study was carried out at my Centre itself, I am fully in agreement with Hong Ye that a large, multicentric randomized clinical trial is required to clarify the role of luteal E2 supplementation on IVF/ICSI outcomes. The other one by Viroj Wiwanitkit on cellular phone and germ cell is also interesting.

And finally I am pleased to say that the ISAR Annual Conference went off really well, was well-attended and greatly appreciated by all delegates. My heartiest congratulations to Dr. Gopinathan, Dr. Fessy Louis and the entire team in Kochi.

